# Factors associated with PEP awareness among adolescent girls and young women in Eswatini

**DOI:** 10.1002/jia2.26486

**Published:** 2025-06-26

**Authors:** Anne Laterra, Stephanie Spaid Miedema, Michelle Li, Phumzile Mndzebele, Nozipho Nzuza‐Motsa, Sana Nasir Charania, Katherine Ong, Meagan Cain, Udhayashankar Kanagasabai, Thobile Mkhonta, Laura Chiang, Francis Boateng Annor, Michelle R. Adler

**Affiliations:** ^1^ Division of Global HIV and Tuberculosis U.S. Centers for Disease Control and Prevention Atlanta Georgia USA; ^2^ Division of Violence Prevention U.S. Centers for Disease Control and Prevention Atlanta Georgia USA; ^3^ Division of Global HIV and Tuberculosis U.S. Centers for Disease Control and Prevention Mbabane Eswatini; ^4^ Eswatini Ministry of Health Mbabane Eswatini; ^5^ U.S. Agency for International Development Mbabane Eswatini

**Keywords:** HIV prevention, adolescent girls and young women, postexposure prophylaxis, Eswatini, Africa, HIV

## Abstract

**Introduction:**

In Eswatini, HIV incidence among adolescent girls and young women (AGYW), aged 15–24 years, is 10 times that of their male peers. Despite the World Health Organization's 2014 recommendation for post‐exposure prophylaxis (PEP) to be available for all HIV exposures, it has been underutilized among youth. PEP is an effective prevention method, and a better understanding of the characteristics, risk factors and behaviours that are associated with PEP awareness, as a precursor to effective use, is needed.

**Methods:**

Using data from the 2022 Eswatini Violence Against Children and Youth Survey, we used logistic regression models to explore the relationships between PEP awareness and a set of hypothesized explanatory variables among AGYW aged 13–24 years who had ever had sex (*N* = 2648). Explanatory variables included socio‐demographic characteristics, sexual risk factors and sexual health behaviours.

**Results:**

A slight majority (57.3%) of AGYW who had ever had sex were aware of PEP as an HIV prevention method. PEP awareness increased with age (aOR 1.1, 95% CI 1.0, 1.1) and was higher among AGYW who had a sexual partner whose age was 5 or more years older in the past 12 months (aOR 1.4, 95% CI 1.1, 1.9), those who had ever taken part in an HIV prevention programme (aOR 1.6, 95% CI 1.2, 2.3) and those who had ever heard of pre‐exposure prophylaxis (aOR 8.1, 95% CI 6.4, 10.2). Participants who were ever married or partnered (aOR 0.7, 95% CI 0.5, 1.0) and those who engaged in inconsistent condom use with non‐spouse/main partner or multiple partners in the past 12 months (aOR 0.8, 95% CI 0.6, 1.00) had lower odds of knowing about PEP in the adjusted model.

**Conclusions:**

We identified sub‐optimal PEP awareness among Swazi AGYW who had ever had sex. Our findings suggest that engagement in HIV prevention programmes increased PEP awareness and that knowing about pre‐exposure prophylaxis (PrEP) was associated with PEP awareness. Future efforts could include tailored PEP awareness activities and campaigns to resonate with AGYW at elevated risk of HIV and integration of PEP education into routine sexual and reproductive service delivery and school‐based HIV curriculum.

## 1 INTRODUCTION

Despite progress in addressing the HIV epidemic, we are not on track to end HIV as a public health threat by 2030 [1]. To reach this goal, we must achieve fewer than 370,000 new HIV acquisitions per year by 2025. In 2022, there were 1.3 million new acquisitions, including 210,000 among adolescent girls and young women (AGYW) aged 15–24 years [2, 3]. In sub‐Saharan Africa, AGYW account for 63% of HIV acquisitions and are three times more likely to acquire HIV than their male peers [2].

In Eswatini, new HIV acquisitions have declined but incidence among AGYW has fallen less rapidly [4, 5]. Women aged 15–24 are nearly 10 times more likely to acquire HIV than men of the same age (incidence of 1.63 per 1000 vs. 0.17 per 1000, respectively) [5]. This context is challenging as 43% of the population is under 18, and high HIV acquisition rates among AGYW threaten effective epidemic control [6].

Awareness of and access to HIV prevention options for those at risk, especially AGYW, are crucial for reducing new acquisitions. Post‐exposure prophylaxis (PEP) for HIV was first introduced in the late 1980s for occupational exposure [7]. In 2014, the World Health Organization updated its guidelines to recommend PEP for all HIV exposures and for all population groups, including adolescents [8].

PEP is currently the only available method that can prevent HIV acquisition following an exposure. An estimated 40% of all new acquisitions in eastern and southern Africa occur among those at medium or low risk [9]. For these lower‐risk individuals, PEP may be a more personally relevant and cost‐efficient HIV prevention method than daily oral PrEP [10]. PEP, as opposed to condoms, is also a user‐controlled prevention method, rather than partner‐controlled, and can be used relatively discretely. Both are important features for AGYW, who may struggle to negotiate condom use and who are often at greater risk of sexual violence [11, 12]. A cluster randomized trial in Uganda and Kenya found that, when given a choice in prevention services, 58% of participants chose PEP at one point during the follow‐up period and that having this choice contributed to a 27.5% increase in antiretroviral‐based prevention coverage [13].

Despite these relative advantages, the limited research to date has found low awareness and use of PEP among youth in sub‐Saharan Africa [14, 15]. Initial analysis of the 2022 Eswatini Violence Against Children and Youth Survey found that less than half (40.4%) of AGYW had heard of PEP [16]. Those who had ever experienced any forced or pressured sex had greater PEP awareness (59%). While awareness does not equate to use, multiple theories of health promotion highlight the importance of knowledge or awareness of a particular health behaviour in determining the adoption of that behaviour [17, 18]. In practice, interventions that incorporate awareness‐raising methods have also been shown to increase PEP use [19]. This analysis aims to understand the factors associated with PEP awareness among AGYW in Eswatini to inform strategies to raise awareness and, ultimately, increase effective PEP utilization. We hypothesize that PEP awareness may differ based on key socio‐demographic characteristics, and that sexual risk factors and health‐seeking behaviours may be positively associated with PEP awareness.

## 2 METHODS

### 2.1 Dataset

We conducted a secondary analysis of select variables from the 2022 Eswatini Violence Against Children and Youth Survey (VACS), a nationally representative cross‐sectional, household‐based survey conducted from April to August 2022 to assess experiences of violence and HIV risk behaviours and health outcomes among adolescents and young people (aged 13–24 years). Full details of the sampling frame, design, eligibility criteria, recruitment and consenting procedures are available elsewhere [16]. The survey used a three‐stage cluster sampling approach, selecting geographic subdivisions as primary sampling units (PSUs). Households within these selected PSUs were listed and pre‐screened for eligibility. Twenty to thirty pre‐screened households in each PSU were selected and one eligible participant per household was randomly selected to participate in the survey. Eligible participants had to be residents of the selected household. Data were collected through an interview‐administered questionnaire covering socio‐demographics; experiences of physical, sexual and emotional violence; sexual history and risk‐taking behaviours; and service seeking and use.

### 2.2 Ethics and consent

This study was approved by the U.S. Centers for Disease Control and Prevention, Columbia University, and Eswatini Health and Human Research institutional review boards (see 45 C.F.R. part 46; 21 C.F.R. part 56) and followed World Health Organization's recommendations on ethics and safety in research on violence against women. Procedures included a youth‐friendly consent process. Data collectors received robust training in responding to disclosures of violence including referral procedures for post‐violence services. A series of checks were in place to ensure the confidentiality and privacy of participants [20, 21].

### 2.3 Measures

#### 2.3.1 Outcome variable

PEP awareness was assessed with the question: “When a person who is HIV‐negative takes pills for 28 days after a single exposure to reduce their chances of getting HIV, this is called post‐exposure prophylaxis or PEP. Have you heard of PEP before now?”. Those who responded “yes” were considered to be aware of PEP, while those responding “no” or “don't know” were considered to be unaware of PEP.

#### 2.3.2 Explanatory variables

Socio‐demographic characteristics included age, education level, marital status and HIV status. Age was measured continuously as age at last birthday. Level of education was assessed based on the highest level of school in which the participant was currently enrolled (for those in school) or had completed (for those out of school) and dichotomized into those currently enrolled or who completed primary or less, and those who were enrolled or completed secondary and higher. Marital status was dichotomized as either ever married or living together or never married or living together as if married. Participants who either self‐reported a prior positive HIV test or tested positive during the survey‐facilitated HIV test were considered HIV positive. Participants who either reported a negative HIV status and refused a survey‐facilitated HIV test or who had a negative survey‐facilitated HIV test were considered HIV negative.

Sexual risk factors included ever having engaged in transactional sex, having multiple sex partners in the prior 12 months, inconsistent condom use, having experienced sexual violence and having a recent sex partner with unknown HIV status. Participants were asked if they ever entered into a sexual relationship with someone “mainly in order to get things that you need such as money, gifts, or other things that are important to you?”. All participants who responded affirmatively were considered to have engaged in transactional sex. Participants were coded as having had multiple sex partners if they reported more than one sexual partner in the 12 months preceding the survey and as having an age‐disparate sex partner if any of their three most recent sexual partners were 5 or more years older than them. Among unmarried participants, inconsistent condom use was coded as never or sometimes using a condom in the past 12 months. Among married/partnered participants, inconsistent condom use was endorsed if the participant had more than one sexual partner (i.e. other sexual partners in addition to their spouse/partner), and never or sometimes used a condom in the past 12 months. Those who endorsed ever experiencing unwanted sexual touching, unwanted forced sex, pressured sex or physically forced sex were considered to have experienced sexual violence. Participants were coded as having had a sex partner with unknown HIV status if they reported not knowing the HIV status of any of their sexual partners in the 12 months prior to the survey.

Sexual health behaviours included HIV testing, sexually transmitted infections (STIs) diagnosis or symptoms, HIV prevention programme participation and access to family planning services. Previous HIV testing was measured by a single question asking participants if they had ever been tested for HIV. Participants were asked if they ever tested positive for a series of STIs (syphilis, gonorrhoea, chlamydia, herpes or genital warts) or if they ever had symptoms of an STI which included unusual discharge, unexplained genital sores or bumps and pain when urinating. Those who reported either a positive STI test or STI symptoms were coded as having had an STI diagnosis or symptom. HIV prevention programme participation was assessed with a single survey item designed in consultation with in‐country stakeholders and included a list of all known PEPFAR or Global Fund‐funded HIV prevention programmes that primarily supported AGYW. Response options included: Insika ya Kusasa, Likusasa Ngeletfu, Phila Unotse, Stepping Stones and DREAMS on Wheels. Participants who reported having ever taken part in at least one of these programmes were considered to have participated. Participants were asked if they or their most recent sex partner used any method to delay or avoid getting pregnant. Those who indicated the use of male sterilization, female sterilization, intrauterine device, injectable, implant, oral contraceptive pill or diaphragm were considered to have accessed a family planning service given all these methods are provided by a healthcare worker.

### 2.4 Analytic procedures

This analysis was conducted among a sub‐sample of VACS participants and included all respondents who provided consent to participate in the survey, were female and had reported ever having had sex (*n* = 2648), which was 41% of the total sample. We assessed awareness of PEP and our hypothesized predictors and checked for co‐linearity between explanatory variables. We estimated the bivariate relationship between explanatory variables and PEP awareness using Pearson chi‐square and *t*‐tests, as appropriate. Finally, we fit a multi‐variate logistic regression model to examine the association between PEP awareness and socio‐demographic characteristics, sexual risk factors and sexual health behaviours. Adequate model fit was assessed using a version of the Hosmer−Lemeshow test appropriate for complex survey data [22]. Listwise deletion was used to account for missing data which was limited to 0.0−2.3% of items included. All analyses accounted for the VACS complex survey design. All analyses were run in StataNow SE/18.5.

## 3 RESULTS

The mean age of AGYW who had ever had sex was 20.5 years with the majority of them having completed or enrolled in secondary school or higher (87.3%) (Table 1). Slightly over 1 in 10 (12.8%) were married or partnered. In the past 12 months, one‐quarter (25.7%) had been in a sexual relationship with someone 5 or more years older, and 10.6% were self‐reported or tested as HIV positive. A minority, 6.8%, had ever engaged in transactional sex. Just under 1 in 10 (7.4%) had had multiple sex partners and had ever experienced sexual violence (13.4%). Almost half (45.8%) had engaged in inconsistent condom use. About one quarter (27.0%) had a sexual partner of unknown or HIV‐positive status in the prior 12 months. Almost all AGYW (97.0%) had ever tested for HIV and more than 1 in 10 (12.5%) had ever been diagnosed with or symptomatic of an STI. Almost one in five AGYW (17.3%) had taken part in an HIV prevention programme. In the past 12 months, one in three (31.8%) had accessed family planning services. Over half (56.7%) had heard of PEP and the majority (70.5%) had heard of PrEP.

**Table 1:** Descriptive statistics of socio‐demographic characteristics, and sexual risk factors and health behaviours among adolescent girls and young women aged 13–24 who had ever had sex in Eswatini, 2022 Eswatini Violence Against Children and Youth Survey (*N* = 2648)

	*N* (mean/weighted %)	[95% CI]
**Socio‐demographic characteristics**		
Age (range: 13–24)	2648 (20.5)	[20.4, 20.6]
Secondary or higher schooling	2306 (87.3)	[85.6, 88.9]
Ever married/partnered	347 (12.8)	[11.3, 14.5]
Age disparate^a^ relationship (past 12 months)	646 (25.7)	[23.4, 28.0]
HIV‐positive status	291 (10.6)	[9.3, 12.1]
**Sexual risk factors**		
Ever engaged in transactional sex	198 (6.8)	[5.6, 8.2]
Multiple sex partners (past 12 months)	185 (7.4)	[6.3, 8.7]
Inconsistent condom use (past 12 months)	1078 (45.8)	[43.2, 48.4]
Ever experienced sexual violence	331 (13.4)	[11.6, 15.5]
Does not know sexual partner HIV status (past 12 months)	543 (23.1)	[21.0, 25.4]
**Sexual health behaviours**		
Previously had tested for HIV	2574 (97.0)	[96.0, 97.8]
Ever diagnosed or symptomatic of STI	326 (12.5)	[10.7, 14.6]
Ever taken part in an HIV prevention programme	430 (17.3)	[15.1, 19.7]
Accessed family planning services in past 12 months	751 (31.8)	[29.2, 34.4]
Had heard of PEP	1528 (56.7)	[53.5, 59.8]
Had heard of PrEP	1882 (70.5)	[67.8, 73.1]

Abbreviations: PEP, post‐exposure prophylaxis; PrEP, pre‐exposure prophylaxis; STI, sexually transmitted infection.

^a^ Sexual relationship with someone 5 or more years older.

At the bivariate level, PEP awareness increased with age (*p*<0.001), and was greater for those who were enrolled or completed secondary school or higher (*p* = 0.006), were in a sexual relationship in the last 12 months with a partner at least 5 years older (*p* = 0.035), were living with HIV (*p* = 0.029), had previously tested for HIV (*p*<0.001), had ever taken part in an HIV prevention programme (*p*<0.001) and had accessed family planning services in the past 12 months (*p* = 0.027). AGYW who did not know the HIV status of a sexual partner in the past 12 months were less likely to have heard of PEP (*p* = 0.048) (Table 2).

**Table 2:** Socio‐demographic characteristics, and sexual risk factors and health behaviours among adolescent girls and young women aged 13–24 who had ever had sex in Eswatini, by awareness of PEP, 2022 Eswatini Violence Against Children and Youth Survey (*N* = 2648)

		Has heard of PEP						
		No		Yes		Total		
	*n*	%/mean (SD)	[95% CI]	%/mean (SD)	[95% CI]	%/mean (SD)	[95% CI]	*p*
**Socio‐demographic characteristics**								
Age (range: 13–24)	2644	20.1 (2.6)	[19.9, 20.3]	20.8 (2.4)	[20.7, 21.0]	20.5 (2.5)	[20.4, 20.7]	<0.001
Secondary or higher schooling	2641	84.8	[81.9, 87.3]	89.5	[87.3, 91.4]	87.5	[85.7, 89.0]	0.006
Ever married/partnered	2633	12.1	[10.0, 14.6]	13.3	[11.3, 15.6]	12.8	[11.3, 14.5]	0.466
Age disparate^a^ relationship (past 12 months)	2386	23.0	[19.9, 26.3]	27.6	[24.7, 30.7]	25.6	[23.4, 28.0]	0.035
HIV‐positive status	2625	9.0	[7.3, 11.0]	11.8	[10.0, 13.9]	10.6	[9.3, 12.1]	0.029
**Sexual risk factors**								
Ever engaged in transactional sex	2626	6.5	[4.7, 8.7]	7.1	[5.6, 8.9]	6.8	[5.6, 8.3]	0.606
Multiple sex partners (past 12 months)	2391	7.2	[5.6, 9.2]	7.6	[6.1, 9.4]	7.4	[6.3, 8.7]	0.776
Inconsistent condom use (past 12 months)	2366	47.8	[44.4, 51.3]	44.4	[40.8, 48.0]	45.8	[43.2, 48.5]	0.171
Ever experienced sexual violence	2642	13.7	[11.1, 16.8]	13.0	[10.8, 15.7]	13.3	[11.5, 15.4]	0.722
Does not know sexual partner HIV status (past 12 months)	2358	25.6	[22.4, 29.1]	21.3	[18.6, 24.3]	23.1	[21.0, 25.4]	0.048
**Sexual health behaviours**								
Previously had tested for HIV	2643	94.7	[92.7, 96.3]	98.9	[98.0, 99.4]	97.1	[96.0, 97.9]	<0.001
Ever diagnosed or symptomatic of STI	2644	11.0	[8.5, 14.1]	13.7	[11.4, 16.4]	12.5	[10.7, 14.6]	0.136
Ever taken part in HIV prevention programme	2639	12.3	[9.7, 15.4]	21.1	[18.1, 24.4]	17.3	[15.1, 19.7]	<0.001
Accessed family planning services (past 12 months)	2391	28.4	[24.7, 32.4]	34.2	[30.8, 37.8]	31.8	[29.2, 34.4]	0.027
Ever heard of PrEP	2640	47.5	[43.9, 51.2]	88.1	[85.8, 90.1]	70.5	[67.8, 73.1]	<0.001

Abbreviations: CI, confidence interval; PEP, post‐exposure prophylaxis; PrEP, pre‐exposure prophylaxis; STI, sexually transmitted infection.

^a^ Sexual relationship with someone 5 or more years older.

After adjusting for all other demographic characteristics, sexual risk factors and sexual health behaviours, PEP awareness was positively associated with participant's age (aOR 1.1, 95% CI 1.0, 1.1), and engagement in age‐disparate sexual relationships in the past 12 months (aOR 1.4, 95% CI 1.1, 1.9). Those who had ever taken part in an HIV prevention programme (aOR 1.6, 95%CI 1.2, 2.3) and those who had ever heard of PrEP (aOR 8.1, 95% CI 6.4, 10.2) had significantly higher odds of having heard about PEP (Table 3). AGYW who were ever married or partnered (aOR 0.7, 95% CI 0.5, 1.0) and those who engaged in inconsistent condom use (aOR 0.7, 95% CI 0.6, 0.9) had significantly lower odds of hearing about PEP.

**Table 3:** Adjusted odds ratios of PEP awareness by socio‐demographic characteristics, and sexual risk factors and health behaviours among adolescent girls and young women aged 13–24 who had ever had sex in Eswatini, 2022 Eswatini Violence Against Children and Youth Survey (*N* = 2301)

	aOR	95% CI	*p‐*value
**Socio‐demographic characteristics**			
Age (range: 13–24)	1.1	[1.0, 1.1]	0.046
Secondary or higher schooling	1.1	[0.8, 1.5]	0.732
Ever married/partnered	0.7	[0.5, 1.0]	0.050
Age disparate^a^ relationship (past 12 months)	1.4	[1.1, 1.9]	0.009
HIV‐positive status	1.2	[0.8, 1.7]	0.418
**Sexual risk factors**			
Ever engaged in transactional sex	1.1	[0.7, 1.8]	0.722
Multiple sex partners (past 12 months)	1.1	[0.7, 1.6]	0.744
Inconsistent condom use (past 12 months)	0.7	[0.6, 0.9]	0.004
Ever experienced sexual violence	1.2	[0.8, 1.7]	0.426
Does not know sexual partner's HIV status (past 12 months)	1.0	[0.8, 1.4]	0.884
**Sexual health behaviours**			
Previously had tested for HIV^a^	1.1	[0.5, 2.9]	0.787
Ever diagnosed or symptomatic of STI	1.2	[0.8, 1.7]	0.346
Ever taken part in HIV prevention programme	1.6	[1.2, 2.3]	0.004
Accessed family planning services (past 12 months)	1.1	[0.8, 1.4]	0.687
Ever heard of PrEP	8.1	[6.4, 10.2]	<0.001
Intercept	0.1	[0.02, 0.31]	<0.001
Model fit, F‐adjusted test statistic (p)	0.411 (0.929)		

Abbreviations: CI, confidence interval; PEP, post‐exposure prophylaxis; STI, sexually transmitted infection.

^a^ Sexual relationship with someone 5 or more years older.

## 4 DISCUSSION

The study assessed PEP awareness and its associations with socio‐demographic characteristics, sexual risk factors and sexual health behaviours among AGYW in Eswatini who had ever had sex. While PEP awareness does not guarantee effective use, it is a necessary precursor to timely access among those who might benefit. Our results demonstrate that PEP awareness is relatively high among this population (57.3%), compared to lower rates of 24% and 25% among university students in South Africa and Nigeria [14, 15]. A qualitative study in western Kenya found that even those aware of PEP lacked a full understanding of the method [23]. Beyond the African continent, PEP awareness varies. In Australia, 21% of university students knew of PEP and a pooled analysis of men who have sex with men found 60% awareness [24, 25]. Although knowledge among AGYW in Eswatini is higher than in some studies, greater awareness is crucial in a context of persistently high incidence and disproportionate risk. Increased awareness of PEP as an available HIV prevention method must be complemented by accurate knowledge of how to access and use PEP effectively. The VACS study found that only about two‐thirds 68.9% of females aware of PEP knew that it must be started within 72 hours [16].

The fully adjusted model indicates that PEP awareness increases with age and is higher among those in age‐disparate relationships, who had ever participated in HIV prevention programming and who were aware of PrEP. The relationship between HIV prevention programme participation and PEP awareness is promising, supporting the theory that such programmes enhance knowledge of HIV prevention methods. One notable initiative, the Determined, Resilient, Empowered, AIDS‐free, Mentored and Safe (DREAMS) programme, has been implemented in approximately 60% of Eswatini's sub‐regions since 2016 and includes educational content to PEP. This may explain the positive relationship between programme participation and PEP awareness.

Even though we found that, in general, knowledge of PEP was lower than knowledge of PrEP among this population, it was encouraging that PrEP and PEP awareness were positively associated. In Eswatini, counselling on PrEP is provided through a variety of service delivery points including HIV testing services, antenatal care clinics and family planning clinics. At these sites, healthcare workers are often trained to identify clients who might benefit from PrEP, counsel or offer PrEP, and in some cases initiate PrEP and provide ongoing support for PrEP use. These investments in PrEP integration may explain why knowledge of PrEP is greater than knowledge of PEP among this population and underscores the importance and value of providing HIV prevention counselling that informs clients about all available prevention methods, including both PrEP and PEP, to promote optimal choice. Interestingly, inconsistent condom use among non‐spouse or multiple partners was associated with a lower likelihood of PEP awareness, which could benefit from further investigation.

While our bivariate analysis identified positive associations between PEP awareness and school attainment, HIV‐positive status, and receipt of HIV testing and family planning services, these associations were not present in the fully adjusted model, suggesting they are attenuated by other factors. Bivariate analyses also indicated a negative relationship between PEP awareness and having a partner with an unknown HIV status, possibly because these participants are less engaged in conversations about HIV and HIV prevention strategies than those who understand their partner's status. Neither the bivariate nor multivariate analyses found evidence of an association between PEP awareness and marital status, engagement in transactional sex, a recent history of multiple sex partners, lifetime experience of sexual violence, or STI diagnosis or symptoms.

Engagement in transactional sex, having multiple recent or concurrent sex partners and having partners with unknown HIV status have all been associated with increased risk of HIV acquisition among AGYW [26, 27, 28, 29]. The lack of or negative associations between these factors and PEP awareness identified in both the bivariate and multivariate analyses suggests that PEP awareness‐building activities and campaigns may benefit from differentiated PEP‐related messages for distinct potential user segments. Key messages could present PEP as an appropriate method for a variety of users including those engaged in transactional sex, those with multiple sex partners, and in sexual relationships with partners with unknown HIV status. Highlighting situations in which PEP might be indicated may help AGYW at risk view PEP as personally relevant and incorporate it as an HIV prevention strategy.

The lack of association between STI diagnosis and treatment in the bivariate and multivariate analyses and between HIV‐positive status, previous HIV testing and receipt of family planning services in the multivariate analysis varied from other prior studies which identified HIV testing as a consistent predictor of higher PEP awareness among youth [14, 15]. These findings suggest that there may be missed opportunities to reach potential PEP users who could be addressed by better integrating PEP awareness building into other sexual and reproductive health services such as HIV testing, STI diagnosis and treatment, family planning services and, for AGYW living with HIV, in care and treatment services as a potential prevention option for their HIV‐negative partners. Eswatini provides free HIV testing services, that allow youth age 12 and above to access testing services in both community and facility settings without requiring parental consent. This includes supporting HIV self‐testing through pharmacies and HIV self‐testing booths. Raising awareness of and linkages to PEP through this strong platform may be an efficient and effective strategy to reach youth.

We had hypothesized that those experiencing sexual violence may have a greater awareness of PEP if they sought services and were counselled on, and perhaps provided with, PEP but no such association was observed, perhaps because post‐violence service seeking is quite low [16]. These findings suggest that integration of PEP awareness building into violence‐prevention approaches and PEP counselling into all post‐violence services, given that those who experience physical violence are often at greater risk of sexual violence, may be a priority area of focus to increase PEP awareness among this vulnerable population. Likewise, the lack of association between level of schooling and PEP awareness in the multivariate model may suggest that there is an opportunity to better integrate PEP awareness into existing or future school‐health or comprehensive sexuality education curricula.

AGYW‐related HIV and other sexual and reproductive health prevention programmes have not been scaled to meet the need [30], and only a relatively modest proportion of this sample reported ever having participated in an HIV prevention programme (17.3%). This means that leveraging other service delivery platforms that reach AGYW such as clinical, pharmacy and education services is a key strategy to expand coverage of PEP education and, ideally, use.

Efforts to increase PEP awareness, knowledge and accessibility in a timely manner could be strengthened by adopting complementary, evidence‐based strategies such as community‐based access and task‐shifting PEP provision to lay health workers or community‐led organizations to ensure PEP is easily available and accessible in a timely manner to those who might need it [13, 31]. Initial studies point to the importance not just of awareness but of PEP availability at a variety of community‐ and facility‐based delivery points and PEP support services such as hot‐lines [19]. Future research is needed to better understand effective strategies to increase awareness of PEP among young people, and how to translate that awareness into effective use, including formative and mixed‐method studies that aim to identify how AGYW prefer to access PEP and be supported in their PEP use.

### 4.1 Strengths and limitations

To our knowledge, this is the first study to examine PEP awareness among a nationally representative sample of AGYW in Africa, and the first analysis of PEP awareness in Eswatini. These exploratory findings can inform the design of PEP communication campaigns and other social behaviour change strategies to increase awareness, and use of PEP among this high‐need group. They also lay the groundwork for understanding barriers and best practices to increase awareness and uptake of PEP, including future analyses that may identify how patterns of these factors might co‐vary to form district segments or audiences that could benefit from tailored PEP‐focused messages.

In terms of limitations, this study focused solely on PEP awareness and did not address use. Future research should explore the relationship between awareness and use and mechanisms for improving PrEP uptake. We also could not include all potential predictors of PEP awareness such as discrimination and stigma, which other studies have linked to knowledge of and access to HIV prevention services [32, 33]. This analysis relied on cross‐sectional data so associations cannot be interpreted as causal. Although trained data collectors administered the survey, social desirability bias may have led participants to overreport PEP awareness and/or underreport stigmatized behaviours. Recall bias may have also impacted the accuracy of self‐reported behaviours. Lastly, these data are from Eswatini, findings may not be applicable across the region or in other contexts and so should be generalized with caution.

## 5 CONCLUSIONS

These findings highlight the suboptimal awareness of PEP among an at‐risk population: AGYW who have ever had sex in Eswatini. Enhancing PEP awareness is a critical step to increasing effective use and complements recommendations to expand access to PEP through community distribution and task‐shifting. The positive association between HIV prevention programme participation and PrEP awareness underscores the effectiveness of these programmes in building knowledge of HIV prevention methods. However, the negative or null associations between PEP awareness and key socio‐demographic characteristics, sexual risk factors and sexual health behaviours, point to (1) gaps in making PEP awareness strategies relevant to those most at‐risk of HIV and (2) missed opportunities for integration of PEP education into routine sexual and reproductive health and education services accessed by youth.

## COMPETING INTERESTS

The authors have no competing interests to declare.

## AUTHORS’ CONTRIBUTIONS

AL: Conceptualization, methodology, writing original draft. SSM: Methodology, formal analysis, writing original draft. ML, SNC, LC and FBA: Methodology, investigation, data curation, project administration, writing review and editing. PM, NN‐M, SNM, KO, MC, UK, TM and MRA: Writing review and editing. The manuscript underwent a review by all authors, and each approved the final version.

## FUNDING

This publication has been supported by the President's Emergency Plan for AIDS Relief (PEPFAR) through the U.S. Centers for Disease Control and Prevention.

## DISCLAIMER

The authors declared no potential conflicts of interest with respect to research, authorship and/or publication of this article. The findings and conclusions in this publication are those of the authors and do not necessarily represent the official position of the funding agencies.

## Data Availability

The public‐use dataset from the 2022 Eswatini Violence Against Children and Youth Survey is available via togetherforgirls.org.

## References

[1] UNAIDS . Describing the ‘end of AIDS as a public health threat’. Geneva: Joint United Nations Programme on HIV/AIDS; 2023.

[2] UNAIDS . The path that ends AIDS: UNIADS Global AIDS Update 2023. Geneva: Joint United Nations Programme on HIV/AIDS: 2023.

[3] UNAIDS . Ending inequalities and getting on track to end AIDS by 2030 — A summary of the commitments and targets within the United Nations General Assembly's 2021 Political Declaration on HIV and AIDS. Geneva: Joint United Nations Programme on HIV/AIDS; 2022.

[4] Government of the Kingdom of Eswatini . Swaziland HIV Incidence Measurement Survey 2 (SHIMS2) 2016–2017. Final Report. Mbabane: Government of the Kingdom of Eswatini; 2019.

[5] Ministry of Health (MOH) . Eswatini Population‐based HIV Impact Assessment 3 2021 (SHIMS32 2021): Final Report. Mbabane: Ministry of Health; 2023.

[6] UNICEF . Country Office Annual Report 2023. Mbabane: UNICEF; 2023.

[7] Public Health Service statement on management of occupational exposure to human immunodeficiency virus, including considerations regarding zidovudine postexposure use. MMWR Recomm Rep. 1990;39(Rr‐1):1–14. 2105451

[8] World Health Organization . Guidelines on post‐exposure prophylaxis for HIV and the use of co‐trimoxazole prophylaxis for HIV‐related infections among adults, adolescents and children: recommendations for a public health approach: December 2014 supplement to the 2013 consolidated guidelines on the use of antiretroviral drugs for treating and preventing HIV infection. Geneva: World Health Organization; 2014. 26042326

[9] Beyrer C , Tomaras GD , Gelderblom HC , Gray GE , Janes HE , Bekker L , et al. Is HIV epidemic control by 2030 realistic? Lancet HIV. 2024;11(7):e489–94. 38925732 10.1016/S2352-3018(24)00098-5 PMC11416730

[10] Maxime Billick K , Tan D , Sapin M , MacFadden D , Bogoch II . HIV PEP‐in‐Pocket (“PIP”) facilities the de‐medicalization of HIV prevention. HIV Research for Prevention. Lima, Peru; 2024.

[11] Duby Z , Jonas K , McClinton Appollis T , Maruping K , Dietrich J , Mathews C . “Condoms are boring”: navigating relationship dynamics, gendered power, and motivations for condomless sex amongst adolescents and young people in South Africa. Int J Sex Health. 2021;33(1):40–57. 38596471 10.1080/19317611.2020.1851334 PMC10807805

[12] World Health Organization . Violence against women prevalence estimates, 2018: global, regional and national prevalence estimates for intimate partner violence against women and global and regional prevalence estimates for non‐partner sexual violence against women. Geneva: World Health Organization; 2021.

[13] Kakande ER , Ayieko J , Sunday H , Biira E , Nyabuti M , Agengo G , et al. A community‐based dynamic choice model for HIV prevention improves PrEP and PEP coverage in rural Uganda and Kenya: a cluster randomized trial. J Int AIDS Soc. 2023;26(12):e26195. 38054535 10.1002/jia2.26195 PMC10698808

[14] Ajayi AI , Yusuf MS , Mudefi E , Adeniyi OV , Rala N , Goon DT . Low awareness and use of post‐exposure prophylaxis among adolescents and young adults in South Africa: implications for the prevention of new HIV infections. Afr J AIDS Res. 2020;19(3):242–248. 33119458 10.2989/16085906.2020.1811356

[15] Ajayi AI , Ismail KO , Adeniyi OV , Akpan W . Awareness and use of pre‐exposure and postexposure prophylaxes among Nigerian university students: findings from a cross‐sectional survey. Medicine (Baltimore). 2018;97(36):e12226. 30200145 10.1097/MD.0000000000012226 PMC6133481

[16] Deputy Prime Minister's Office . Violence against children and youth, Kingdom of Eswatini: findings from the Violence Against Children and Youth Survey, 2022 (Final Report). Mbabane: Government of the Kingdom of Eswatini; 2023.

[17] Schunk D . Social cognitive theory. American Psychological Association; 2012.

[18] Prochaska J , Velicer W . The transtheoretical model of health behavior change. Am J Health Promot. 1997;12(1):38–48. 10170434 10.4278/0890-1171-12.1.38

[19] Ayieko J , Petersen ML , Kabami J , Mwangwa F , Opel F , Nyabuti M , et al. Uptake and outcomes of a novel community‐based HIV post‐exposure prophylaxis (PEP) programme in rural Kenya and Uganda. J Int AIDS Soc. 2021;24(6):e25670. 34152067 10.1002/jia2.25670 PMC8215805

[20] Chiang LF , Kress H , Sumner SA , Gleckel J , Kawemama P , Gordon RN . Violence Against Children Surveys (VACS): towards a global surveillance system. Inj Prev. 2016;22(Suppl 1):i17–22. 27044493 10.1136/injuryprev-2015-041820 PMC6158784

[21] Centers for Disease Control and Prevention . Critical elements of interviewer training for engaging children and adolescents in global violence research: best practices and lessons learned from the Violence Against Children Survey. Atlanta, GA: National Center for Injury Prevention and Control, Centers for Disease Control and Prevention; 2017.

[22] Archer K , Lemeshow S . Goodness‐of‐fit test for a logistic regression model fitted using survey sample data. Stata J. 2006;6(1):97–105.

[23] Miller L , Otieno B , Amboka S , Kadede K , Odeny D , Odhiambo H , et al. “Something like that”: awareness and acceptability of HIV PrEP and PEP among Kenyan adolescents. Int J Behav Med. 2024. Online ahead of print. https://pubmed.ncbi.nlm.nih.gov/38942977/. 10.1007/s12529-024-10290-6 38942977

[24] Warzywoda S , Dyda A , Fitzgerald L , Mullens A , Debattista J , Durham J , et al. A cross‐sectional investigation of the factors associated with awareness of PEP and PrEP among Queensland university students. Aust N Z J Public Health. 2024;48(2):100136. 38432178 10.1016/j.anzjph.2024.100136

[25] Jin J , Sun R , Mu T , Jiang T , Dai L , Lu H , et al. Awareness and use of post‐exposure prophylaxis for HIV prevention among men who have sex with men: a systematic review and meta‐analysis. Front Med (Lausanne). 2021;8:783626. 35083243 10.3389/fmed.2021.783626 PMC8784556

[26] Low A , Thin K , Davia S , Mantell J , Koto M , McCracken S , et al. Correlates of HIV infection in adolescent girls and young women in Lesotho: results from a population‐based survey. Lancet HIV. 2019;6(9):e613–22. 31422056 10.1016/S2352-3018(19)30183-3 PMC6829164

[27] Mabaso M , Sokhela Z , Mohlabane N , Chibi B , Zuma K , Simbayi L . Determinants of HIV infection among adolescent girls and young women aged 15–24 years in South Africa: a 2012 population‐based national household survey. BMC Public Health. 2018;18(1):183. 29373958 10.1186/s12889-018-5051-3 PMC5787232

[28] Santelli JS , Edelstein ZR , Mathur S , Wei Y , Zhang W , Orr MG , et al. Behavioral, biological, and demographic risk and protective factors for new HIV infections among youth in Rakai, Uganda. J Acquir Immune Defic Syndr. 2013;63(3):393–400. 23535293 10.1097/QAI.0b013e3182926795 PMC4131841

[29] Rucinski KB , Mbita G , Atkins K , Majani E , Komba A , Casalini C , et al. Transactional sex and age‐disparate sexual partnerships among adolescent girls and young women in Tanzania. Front Reprod Health. 2024;6:1360339. 39055125 10.3389/frph.2024.1360339 PMC11269161

[30] UNAIDS . World AIDS Day 2023 Fact Sheet. Geneva; 2023.

[31] World Health Organization . Guidelines for HIV post‐exposure prophylaxis. Geneva: World Health Organization; 2024. 39259822

[32] Velloza J , Khoza N , Scorgie F , Chitukuta M , Mutero P , Mutiti K , et al. The influence of HIV‐related stigma on PrEP disclosure and adherence among adolescent girls and young women in HPTN 082: a qualitative study. J Int AIDS Soc. 2020;23(3):e25463. 32144874 10.1002/jia2.25463 PMC7060297

[33] Logie CH , Okumu M , Mwima SP , Kyambadde P , Hakiza R , Kibathi IP , et al. Exploring associations between adolescent sexual and reproductive health stigma and HIV testing awareness and uptake among urban refugee and displaced youth in Kampala, Uganda. Sex Reprod Health Matters. 2019;27(3):86–106. 31880507 10.1080/26410397.2019.1695380 PMC7888033

